# Metabolic Changes in SARS-CoV-2 Infection: Clinical Data and Molecular Hypothesis to Explain Alterations of Lipid Profile and Thyroid Function Observed in COVID-19 Patients

**DOI:** 10.3390/life11080860

**Published:** 2021-08-22

**Authors:** Damiano D’Ardes, Ilaria Rossi, Benedetta Bucciarelli, Marco Allegra, Francesco Bianco, Bruna Sinjari, Michele Marchioni, Marta Di Nicola, Francesca Santilli, Maria Teresa Guagnano, Francesco Cipollone, Marco Bucci

**Affiliations:** 1 “Clinica Medica” Institute, Department of “Medicine and Science of Aging”, “G. d’Annunzio” University, 66100 Chieti, Italy; benedetta.bucciarelli@studenti.unich.it (B.B.); marco.allegra@studenti.unich.it (M.A.); francesca.santilli@unich.it (F.S.); guagnano@unich.it (M.T.G.); francesco.cipollone@unich.it (F.C.); mbucci@unich.it (M.B.); 2 Azienda Sanitaria Locale n° 2 Abruzzo Lanciano-Vasto-Chieti, 66100 Chieti, Italy; 3 Pediatric Cardiology and Adult Congenital Heart Disease, Azienda Ospedaliero-Universitaria “Ospedali Riuniti” of Ancona, 60126 Ancona, Italy; francesco.bianco@ospedaliriuniti.marche.it; 4 Department of Innovative Technologies in Medicine and Dentistry, “G. d’Annunzio” University, Chieti-Pescara, 66100 Chieti, Italy; b.sinjari@unich.it; 5 Laboratory of Biostatistics, Department of “Medical, Oral and Biotechnological Sciences”, “G. d’Annunzio” University, Chieti-Pescara, 66100 Chieti, Italy; michele.marchioni@unich.it (M.M.); mdinicola@unich.it (M.D.N.)

**Keywords:** COVID-19, SARS-CoV-2, lipids, cholesterol, thyroid

## Abstract

It seems that during SARS-CoV-2 infection, total cholesterol, LDL-C, and HDL-C values decrease and lipids could play a fundamental role in viral replication. Moreover, it has been shown that SARS-CoV-2 infection could influence thyroid function. We performed a retrospective analysis of 118 hospitalized patients with COVID-19, comparing pre-infection lipid profile (53 patients) and thyroid-stimulating hormone (TSH) values (45 patients) to those measured on admission. Our aim was to evaluate whether SARS-CoV-2 infection could be involved in thyroid and lipid profile alterations and study possible correlations with disease severity and clinical outcome. Median baseline values at the admission time were: total cholesterol at 136.89 ± 42.73 mg/dL, LDL-C 81.53 ± 30.35 mg/dL, and HDL-C 32.36 ± 15.13 mg/dL; and triglycerides at 115.00 ± 40.45 mg/dL, non-HDL-C 104.53 ± 32.63 md/dL, and TSH 1.15 ± 1.08 μUI/mL. Median values of pre-infection total cholesterol, HDL-C, and TSH were significantly higher than those measured at the admission time (*p* value < 0.05). The C-reactive protein (CRP) negatively correlated with LDL-C (*p* = 0.013) and HDL-C (*p* = 0.05). Our data underline a possible impact of SARS-CoV-2 infection on thyroid function. Moreover it suggests a possible relation between COVID-19 and the lipid profile with a negative correlation between CRP, LDL-C, and HDL-C values, proposing the hypothesis that lipid lowering could follow the rising of the COVID-19 inflammatory state.

## 1. Introduction

Coronavirus disease 2019 (COVID-19) is, at present, one of the most relevant global health problems caused by severe acute respiratory syndrome coronavirus 2 (SARS-CoV-2), a single-stranded RNA virus belonging to the Betacoronavirus genus [1].

The first cases of COVID-19 were identified in December 2019 in Wuhan, Hubei province of China, where an increase in access to local hospitals of adults with pneumonia of unknown etiology was observed [2,3]. Respiratory samples of patients were sent to reference labs for etiologic investigations after activation of a surveillance system and on 31 December 2019 China, notified the outbreak to the World Health Organization (WHO) [2]. Due to the following worldwide spread of the infection, the WHO declared COVID-19 as the sixth public health emergency of international concern on 30 January 2020 and as a pandemic on 11 March 2020 [4].

Among the alterations caused by COVID-19, those of lipid metabolism have been poorly investigated to date. Some studies have demonstrated that COVID-19 patients have decreased LDL-C, HDL-C, and total cholesterol levels [5,6].

In particular, it has recently been shown that LDL-C levels decreased at the time of hospital admission, remained low during the treatment, and returned to the levels prior to infection at discharge [5].

The same trend could be observed for total cholesterol, while the HDL-C levels after the initial reduction at the time of admission remained low not only during the treatment but also after recovery [5].

Interestingly, the LDL-C, HDL-C, and total cholesterol levels in non-surviving patients continuously decreased until death. It has also been shown that HDL-C levels decrease only in the most critically ill patients and that also patients with mild symptoms can develop hypolipidemia with a degree that positively correlates with the severity of the disease [6,7].

Moreover LDL-C cholesterol can be considered as a potential predictor of poor prognosis in patients with COVID-19 [5,6]. 

Concerning other aspects of metabolism, it has been shown that SARS-CoV-2 infection could play a role in thyroid function.

In fact, even if most patients with COVID-19 present with euthyroidism, mild reductions in the thyroid-stimulating hormone (TSH) and free thyroxin (FT)4 have been observed [8].

The thyroid gland and the virus infection with its associated inflammatory-immune responses are known to be engaged in a complex interplay [9].

In light of these data, we performed a retrospective analysis of patients’ TSH values and lipid profile, comparing pre-infection values to those measured during hospitalization for COVID-19, with the aim of evaluating whether significant changes occurred and if they could be related to the severity of the pathology and prognosis.

## 2. Materials and Methods

We performed a retrospective study on 118 patients admitted for COVID-19 to our Internal Medicine Unit of Chieti University Hospital (Italy) between 18 March and 30 April 2020. 

We assessed the trend of TSH values and of the lipid profile, including the total cholesterol, HDL-C, and tryglicerides (TG), at the time of admission. Then, we surfed our laboratory exams software to search previous values of TSH, total cholesterol, HDL-C, and TG measured on blood samples routinely drawn from the same patients before SARS-CoV-2 infection at a minimum distance of three months and maximum of three years before the hospitalization for COVID-19, with an average time of one year prior to infection. LDL-C and non-HDC-C were calculated indirectly; for LDL-C we used the Friedewald formula [LDL-C = total cholesterol – (HDL-C + (TG/5)] [10]. We also evaluated if and how TSH, LDL-C, HDL-C, and total cholesterol levels correlated with the clinical outcome and disease severity. Patients in home therapy for dyslipidemia and thyroid disease were not excluded as the therapy taken at the time of pre-admission laboratory tests and at the time of admission was the same.

The diagnosis of COVID-19 was made through a nasopharyngeal swab using a real-time reverse transcription polymerase chain reaction. Data regarding epidemiological, demographic, clinical symptoms, diagnosis, laboratory, and radiological tests and treatment were extracted from the medical record. The laboratory exams were collected within 24 h from admission. These exams were not influenced by hospital therapies used to treat COVID-19 patients according to the scientific evidence available at the moment of the pandemic. World Health Organization criteria were used to define COVID-19 severity [11]. The study was performed in accordance with the principles embodied in the Declaration of Helsinki.

Quantitative variables were summarized as the median ± standard deviation and interquartile range (IQR), while qualitative data were reported as frequency and percentage. Deviations from normal distribution were evaluated for each variable using a Shapiro—Wilk’s test.

Linear mixed models were fitted to test the effect of patients’ characteristics (as independent variables) on the clinical and microbiological time to recovery. The Wilcoxon test was used to compare differences between groups. 

All tests were two-sided and the statistical significance level was set at *p* < 0.05. All the statistical analyses were performed using the R software environment for statistical computing and graphics version 3.5.2 (R Foundation for Statistical Computing, Vienna, Austria. https://www.R-project.org/; accessed on 13 June, 2020).

## 3. Results

The cohort of 118 patients (Table 1) showed on admission: a total cholesterol 143.59 ± 40.45 mg/dL (115 patients, IQR: 114.00–169.00 mg/dL), LDL-C 86.88 ± 29.90 mg/dL (113 patients, IQR: 64.6–103.90 mg/dL), and HDL-C 33.78 ± 15.41 mg/dL (113 patients, IQR: 25.00–37.50 mg/dL); triglycerides 114.48 ± 48.41 mg/dL (114 patients, IQR: 80.75–137.75 mg/dL); and non-HDL-C 109.81 ± 32.85 mg/dL (113 patients, IQR: 85.00–130.50 mg/dL). 

Looking for previous values of the lipid profile, we found a subcohort of 53 patients that had a routine laboratory exam collected in our hospital before SARS-CoV-2 infection. In particular, this subcohort showed on admission: a total cholesterol of 136.89 ± 42.73 mg/dL (IQR: 109.50–157.50 mg/dL), LDL-C 81.53 ± 30.35 mg/dL (IQR: 57.90–98.90 mg/dL), and HDL-C 32.36 ± 15.13 mg/dL (IQR: 23.00–34.50 mg/dL); triglycerides 115.00 ± 40.45 mg/dL (IQR: 85.50–139.00 mg/dL); and non-HDL-C 104.53 ± 32.63 md/dL (IQR: 77.00–123.00 mg/dL). 

The median subcohort pre-infection values were: for total cholesterol 158.43 ± 45.18 mg/dL (IQR: 122.00–191.00 mg/dL), LDL-C 90.16 ± 34.44 mg/dL (IQR: 61.80–114.30 mg/dL), and HDL-C was 44.09 ± 17.76 mg/dL (IQR: 31.00–58.00 mg/dL); tryglicerides 121.00 ± 59.09 mg/dL (IQR: 83.50–147.50 mg/dL); and non-HDL-C 114.36 ± 39.30 mg/dL (IQR: 82.50–142.00 mg/dL). In particular, total cholesterol and HDL-C pre-infection values were significantly higher than those measured during the acute phase of SARS-CoV-2 infection (respectively *p =* 0.001 and *p* < 0.001), suggesting the possible existence of a physiopathological link between SARS-CoV-2 infection and the alteration of plasma lipids’ concentrations. All data are reported in Table 2.

Interestingly, the C-reactive protein (CRP) negatively correlated with LDL-C (*p =* 0.013) and HDL-C, even if at the limit of statistical significance with *p =* 0.05, suggesting that the lipid profile lowering could follow the rising of the inflammatory state typical of COVID-19. However, we found no correlation between the plasma lipids lowering and the disease severity, nor clinical outcome.

Concerning the thyroid profile, the median TSH for 115/118 patients at admission was 1.14 ± 1.46 μUI/mL (IQR: 0.46 μUI/mL–1.34 μUI/mL). In this patient group, the pre-admission TSH value was available in a subcohort of 45 subjects (Table 3): the median TSH at admission for these 45 patients was 1.15 ±1.08 μUI/mL (IQR: 0.52 μUI/mL–1.29 μUI/mL) and the pre-infection TSH value was 1.67 ± 1.67 μUI/mL (IQR: 1.23 μUI/mL–2.09 μUI/mL). 

The difference between the pre-admission TSH and the value detected at the time of acute SARS-CoV-2 infection was statistically significant (*p =* 0.014), suggesting an influence of COVID-19 on thyroid function. 

## 4. Discussion 

Our retrospective analysis of the thyroid and lipid profile in COVID-19 patients proves that patients with SARS-CoV-2 infection develop hypolipidemia with reduced levels of LDL-C that negatively correlated with CRP and that TSH levels also appear to be reduced. 

In comparing pre-infection values with those detected during hospitalization for COVID-19, a statistically significant reduction of total cholesterol and HDL-C values emerged. A decrease in LDL-C and non-HDL-C levels was also noted, even if not statistically significant.

Concerning the relation between lipids and COVID-19, it is important to remember that viruses are intracellular parasites that have to use the human metabolic system to satisfy their biosynthetic needs. 

The relationship between lipids and SARS-CoV-2 is very interesting. In fact, in in vitro, this virus seems to modulate the lipid metabolism in human monocytes and has also shown an up-regulation by SARS-CoV-2 of the lipid metabolism and lipid droplets’ biogenesis, involving organelles with major functions in lipid metabolism, energy homeostasis, and intracellular transport, and have multiple roles in infections and inflammation: this suggests that the virus may explore the host metabolism to favor its replication, using the lipid droplets as a replication platform [12]. 

In addition, viral internalization is guided by lipids through the invagination of the plasma membrane: thus, viral entry is dependent on the attachment and fusion of the viral membrane with the plasma membrane through an endocytosis-mediated process, and the role of lipid rafts in the viral entry into the host cells is of great relevance [13]. 

Other authors have already discussed the link between COVID-19 and lipidic metabolism [5,6]: a possible responsible factor may be the liver damage that could occur during COVID-19. The researchers who hypothesized a possible correlation between impaired liver function and hypolipidemia described an increase, although mild or moderate, in transaminases in about half of all patients with COVID-19 [5,6]. As a consequence, the liver damage induced by SARS-CoV-2 could reduce LDL-C biosynthesis, but this hypothesis has not been demonstrated yet. 

Inflammation itself could in fact play an important role in lipid metabolism alteration. Viral infections are known to be associated with dyslipidemia: this has been observed in bacterial infection and in acute and chronic viral infections (such as HIV, HBV, and HCV infections) [14]. In particular, HIV viremia is associated with low HDL-C, low LDL-C, and high triglycerides [15]. 

Concerning SARS-CoV-2 infection, it has recently been shown that IL-6 dramatically increased in 96% of all patients investigated, suggesting that proinflammatory cytokines and therefore acute inflammation in general are major contributors to the altered lipid metabolism in COVID-19 patients [6]. 

In relation to HIV infections, it has been reported that proinflammatory cytokines, including IL-6, can modulate lipid metabolism by altering liver function and reducing cholesterol efflux and transport [15]. 

We found a significant correlation between LDL-C levels and CRP, as also other authors showed [5,6]. In particular, high CRP values were found to be associated with low LDL-C and HDL-C values as well as high TG values.

In addition to liver damage and inflammation, the lipid degradation favored by free radicals, which are generally increased in host cells with a viral infection [16,17], could help to explain, at least in part, the lipid profile alterations as we observed in the cohort we studied. 

A final hypothetical mechanism linking the lipid profile and COVID-19 concerns the altered vascular permeability induced by SARS-COV-2 infection, which can lead to the accumulation of exudative fluids in the alveoli [5,6]. As effusion contains proteins and cholesterol [18,19,20], the cholesterol exudate accumulating in the alveolar cavities due to the increase in vascular permeability could lead to a reduction in serum cholesterol levels. 

Several studies have already described postmortem findings of SARS [21], identifying diffuse alveolar damage (DAD) as the primary cause of respiratory failure [22,23]. Autopsies of COVID-19 patients showed that the most prominent histological findings were severe capillary congestion, hyaline membranes, reactive pneumocyte changes, and exudative DAD [22]. 

Zhang et al. showed the in vitro down-regulation of reverse cholesterol transport due to ABCA1 suppression in both HepG2-cells and THP-1-derived macrophages when exposed to IL-6 and TNF-α [24]. Thus, hypolipidemia detected in COVID-19 patients showing a hyperinflammation condition could be due to liver damage and reverse cholesterol transport suppression. As a consequence, Wang et al. proposed a possible pathological pathway for SARS-CoV-2 that could create an intracellular environment ideal for its biological success, especially in pneumocytes [25,26]. 

Finally, the role of s SREBP-2 is emerging as disturber of cholesterol biosynthesis and activator of cytokine storm [27]. Sterol regulatory element binding proteins (SREBPs) regulate the lipid cholesterol and fatty acid gene expressions promoting the expression of cholesterogenic and lipogenic genes [27]. In particular, SREBP-2 is known to be a transcription factor for lipid synthesis, but in COVID-19 patients, the level of cholesterol is maintained in a low level, although the expression of SREBP-2 is increased [27]. Recently, it has been reported that SREBP-2-mediated biosynthesis of cholesterol is involved in the exocytosis process of SARS-CoV2, which explains its role in virus budding and envelop [27]. The SREBP-2 C-term fragment in these patients also correlated with inflammatory cytokine release and vascular disruption and has therefore been hypothesized as a therapeutic target [27].

Concerning thyroid data, it is important to underline that SARS-CoV-2 uses the ACE2 receptor associated to transmembrane protease serine 2 (TMPRSS2) as the molecular key to infect human cells [28]. Moreover, ACE2 and TMPRSS2 expression levels are high in the thyroid gland and even more than in the respiratory tract [9]. 

Regarding this aspect, in silico studies also show that the thyroid ACE2 levels are linked to immune signatures, contributing to the explanation of the different immune responses and the resultant distinct thyroid manifestations [29]. 

Uptake by host cells of SARS-CoV-2 is secondarily thought to involve other cellular molecules and proteases as well as integrins, which are amongst the most important proteins involved in the cell invasion of SARS-CoV-2 [30]. 

ACE2 binds to integrin modulating signal transduction [31]. It is important to underline that thyroid hormones modulate the expression of genes coding for the monomeric protein that makes up integrins and thyroid hormones are thought to encourage the internalization of the integrins [31]. As a consequence, thyroid hormones could positively influence the SARS-CoV-2 uptake involving integrins [31].

Moreover, SARS-CoV-2 could indirectly affect the thyroid considering the cytokine storm linked to COVID-19 is a possible trigger perpetuating thyroid gland inflammation [9,32].

Alongside the pathophysiological hypotheses, our study confirms incontrovertible clinical data. In fact, we could affirm that our study is most likely one of the first comparing TSH and lipid profile values measured before and after SARS-CoV-2 infection in the same patients, collecting blood samples right on admission, thus not being modified or influenced by hospital care therapies. Either way, our study also presents some limitations. It is worthy to note that it was not possible to find a lipidic and thyroid assessment prior to hospitalization for all patients; thus, the study population was reduced in comparison to our expectations and, unfortunately, free thyroid hormone fractions have not been collected in the patients. 

Moreover, the time from the onset of symptoms was different among patients at the moment of serum sample collection on admission, thus creating a cohort of patients with different stages of the disease course. Finally, we were not able to strictly monitor the dynamics of the lipid and thyroid profiles during the entire disease course. 

However, even if limited in terms of numerosity, our data underlines a fundamental role of lipids and their laboratoristic changes in SARS-CoV-2 infection with possible relevant clinical consequences. 

Our data also underlines the influence of COVID-19 on thyroid function, revealing an intriguing clinical connection that could have found its foundation on a molecular basis.

## 5. Conclusions

Our findings showed a reduction of total cholesterol, HDL-C, and TSH values in patients with COVID-19. Moreover, LDL-C and HDL-C seem to become lower as inflammation rises. The pathological pathway leading to hypolipidemia found in COVID-19 patients is not yet known but inflammation may be the predominant cause. 

Thanks to our observations and the recent literature available, we can emphasize that SARS-CoV-2 infection has an impact on thyroid function and on lipid profile, but more data are needed to better understand the pathological mechanisms and clinical consequences. 

## Figures and Tables

**Table 1:** General and clinical characteristics of all patients (118), with specific detail on the subcohort of patients with (53) and without (65) the previous pre-admission of the lipid profile and subcohort of patients with (45) and without (73) previous TSH.

General and Clinical Characteristics of Patients					
Characteristics	All Patients (118)	Patients with Pre-Admission Lipid Profile (53)	Patient without Pre-Admission Lipid Profile (65)	Patients with Pre-Admission TSH (45)	Patients without Pre- Admission TSH (73)
	Median ± Standard Deviation				
Age (years)	72.95 ± 17.26	70.00 ± 13.77	68.83 ± 18.78	75.29 ± 16.69	71.51 ± 17.56
Days of hospitalization	19.19 ± 11.68	17.43 ± 11.23	20.62 ± 11.93	18.04 ± 12.51	19.89 ± 11.17
Characteristics	**Patients Number (percentage)**				
**Sex**					
Male	64 (54.2)	31 (58.5)	33 (50.8)	21 (46.7)	43 (58.9)
Female	54 (45.8)	22 (41.5)	32 (49.2)	24 (53.3)	30 (41.1)
**Comorbidities**					
COPD	12 (10.2)	8 (15.1)	4 (6.2)	6 (13.3)	6 (8.2)
Diabetes mellitus	14 (11.9)	10 (18.9)	4 (6.2)	7 (15.6)	7 (9.6)
Obesity	8 (6.8)	4 (7.5)	4 (6.2)	5 (11.1)	3 (4.1)
Hypertension	70 (59.3)	37 (69.8)	33 (50.8)	31 (70.5)	39 (53.4)
Dyslipidemia	21 (17.8)	14 (26.4)	7 (10.8)	9 (20.0)	12 (16.4)
Thyroid disease	12 (10.2)	4 (7.5)	8 (12.3)	7 (15.6)	5 (6.8)
Paroxysmal Atrial fibrillation	7 (5.9)	3 (5.7)	4 (6.2)	4 (8.9)	3 (4.1)
Chronic atrial fibrillation	16 (13.6)	8 (15.1)	8 (12.3)	8 (17.8)	8 (11.0)
Heart failure	34 (28.8)	21(39.6)	13 (20.0)	18 (40.0)	16 (21.9)
Ischemic heart disease	21 (17.8)	16 (30.2)	5 (7.7)	12 (26.7)	9 (12.3)
Active cancer	5 (4.2)	3 (5.7)	2 (3.1)	4 (8.9)	1 (1.4)
Previous cancer	11 (9.3)	7 (13.2)	4 (6.2)	5 (11.1)	6 (8.2)
**Therapies**					
Lipid lowering therapy	12 (10.2)	7 (13.2)	5 (7.7)	5 (11.1)	7 (9.6)
Therapy for thyroid disease	9 (7.6)	4 (7.5)	5 (7.7)	5 (11.1)	4 (5.5)
**COVID-19 Symptoms and Outcome**					
Fever	92 (78%)	40 (75.5)	52 (80)	33 (73.3)	59 (80.8)
Dyspnea	51 (44.3)	23 (43.4)	28 (43.1)	21 (50.0)	30 (41.1)
Desaturation	55 (46.6)	24 (45.3)	31 (47.7)	20 (44.4)	35 (47.9)
Death	26 (22)	17 (32.1)	9 (13.9)	14 (31.1)	12 (16.4)
**Severity**					
Asymptomatic	0	0	0	0	0
Mild symptoms	4 (3.4)	2 (3.8)	2 (3.1)	3 (6.7)	1 (1.4)
Mild pneumonia	71 (60.2)	25 (47.2)	46 (70.8)	23 (51.1)	48 (85.8)
Severe pneumonia	42 (35.6)	26 (49.1)	16 (24.6)	19 (42.2)	23 (31.5)
ARDS	1 (0.8)	0	1 (1.5)	0	1 (1.4)

**Table 2:** Total cholesterol, LDL-C, HDL-C, and TG values measured on admission to the Internal Medicine Unit of Chieti University Hospital and comparison with pre-infection corresponding values in the subcohort of 53 patients.

	Before SARS-CoV-2 Infection mg/dL (IQR mg/dL)	On Admission for COVID-19 mg/dL (IQR mg/dL)	*p*-Value
Total cholesterol	158.43 ± 45.18 (122.00–191.00)	136.89 ± 42.73 (109.50–157.50)	0.001
LDL-C	90.16 ± 34.44 (61.80–114.30)	81.53 ± 30.35 (57.90–98.90)	0.101
HDL-C	44.08 ± 17.76 (31.00–58.00)	32.36 ± 15.13 (23.00–34.50)	0.000
TG	121.00 ± 59.09 (83.50–147.50)	115.00 ± 40.45 (85.50–139.00)	0.378
non-HDL-C	114.36 ± 39.30 (82.50–142.00)	104.53 ± 32.63 (77.00–123.00)	0.060

Abbreviations: LDL-C, low density lipoproteins, values calculated with Friedewald formula; HDL-C, high density lipoproteins; TG, triglycerides; and non-HDL-C, non-high-density lipoprotein cholesterol. *p* values accepted when *p* < 0.05.

**Table 3:** TSH values measured on admission to the Internal Medicine Unit of Chieti University Hospital and comparison with pre-infection corresponding values on 45 patients.

	Before SARS-CoV-2 Infection mg/dL (IQR mg/dL)	On Admission for COVID-19 mg/dL (IQR mg/dL)	*p* value
TSH	1.67 ± 1.67 (IQR: 1.23–2.09)	1.15 ± 1.08 (IQR: 0.52–1.29)	0.014

*p* values accepted when *p* < 0.05.

## Data Availability

The data presented in this study are available on request from the corresponding author.
